# Quantitative assessment of muscle fatigue during rowing ergometer exercise using wavelet analysis of surface electromyography (sEMG)

**DOI:** 10.3389/fbioe.2024.1344239

**Published:** 2024-02-28

**Authors:** Natalia Daniel, Jerzy Małachowski, Kamil Sybilski, Dariusz Siemiaszko

**Affiliations:** ^1^ Institute of Rocket Technology and Mechatronics, Faculty of Mechatronics, Armament and Aviation, Military University of Technology, Warsaw, Poland; ^2^ Institute of Mechanics & Computational Engineering, Faculty of Mechanical Engineering, Military University of Technology, Warsaw, Poland; ^3^ Department of Functional Materials and Hydrogen Technology, Military University of Technology, Warsaw, Poland

**Keywords:** electromyography, EMG, rowing ergometer, wavelet transform, DWT

## Abstract

In this paper, we present a quantitative assessment of muscle fatigue using surface electromyography (sEMG), a widely recognized method that is conducted through various analytical approaches, including analysis of spectral and time-frequency distributions. Existing research in this field has demonstrated considerable variability in the computational methods used. Although some studies highlight the efficacy of wavelet analysis in dynamic motion, few offer a comprehensive method for determining fatigue and applying it to specific movements. Previous research has focused primarily on discerning differences based on sport type or gender, with a notable absence of studies that presented results for quantifying fatigue during exercise with rowing ergometers. Developing on our previous work, where we introduced a method for determining muscle fatigue through wavelet analysis, considering biomechanical aspects of limb position changes, this current article serves as a continuation. Our study refines the research approach for a selected group, focusing on fatigue determination using the previously established method. The results obtained confirm the effectiveness of DWT analysis in assessing muscle fatigue, as evidenced by the achievement of negative values of the regression coefficients of Median Frequency (MDF) during exercises performed to maximal fatigue. Furthermore, it has been confirmed that the homogeneity of the group and, in the case of the examined group, the results previously achieved or lower limb strength do not have an impact on the results. Finally, we discuss the main limitations of our study and outline the subsequent steps of our investigation, providing valuable information for future investigations in this field.

## 1 Introduction

Electromyography (EMG) is a method of measuring electrical signals generated in skeletal muscles to quantitatively assess muscle fatigue or exertional force (Hou et al., 2021; Son et al., 2022). Quantitative assessment of muscle fatigue using surface electromyography (sEMG) has been the subject of numerous studies (Zhang et al., 2019; Yun et al., 2020a; Yun et al., 2020b) leading to the need to develop electromyographic models that can correlate changes in sEMG signals with muscle fatigue (González-Izal et al., 2012). Evaluation of muscle fatigue based on EMG signals can be performed using various methods, including spectral analysis, time-frequency distribution analysis, fractal analysis, and signal entropy analysis (González-Izal et al., 2012; Sun et al., 2022). One of the most popular methods for quantitatively determining muscle fatigue based on non-invasive sEMG measurements is spectral analysis, specifically analyzing the frequency parameters: mean frequency (MNF) and median frequency (MDF) of the electromyographic signal (Phinyomark et al., 2012a; Kim et al., 2020). Numerous studies have shown that an increase in muscle fatigue leads to a decrease in the values of MDF and MNF over time (Dantas et al., 2010; Yousif et al., 2019a; Özgören and Arıtan, 2022). This is attributed to the relationship between these frequency parameters and changes in muscle fiber conduction velocity (González-Izal et al., 2012).

Research on the quantitative assessment of muscle fatigue encompasses both dynamic and static movements (Masuda et al., 1999; Wang et al., 2007; Wan et al., 2010; Chowdhury and Nimbarte, 2017). In one study, the indirect use of the MDF parameter as an indicator of local muscle fatigue during static arm movements was discussed (Kuthe et al., 2018). The study demonstrated, among other findings, that the mean slope of the linear regression of MDF for the untrained group (−0.2470) was lower than that of the trained group (−0.2155). Together with force measurements, the authors concluded that applied EMG analysis could be used as a quantitative indicator of fatigue in static muscle contractions (Kuthe et al., 2018). In the field of dynamic movement research, it has been shown, for example, that the slope of the linear regression of MDF determined by Fast Fourier Transform (FFT) analysis for the lateral vastus muscle during dynamic elliptical exercises is lower for women (−0.0224) than for men (−0.0170). Additionally, no correlation was found between sEMG parameters and strength levels (Chang et al., 2012). Another intriguing example from dynamic movement research involved determining the values of linear regression coefficients for MDF and MNF using FFT analysis for muscles: Rectus Femoris (RF), Biceps Femoris (BF), and Gastrocnemius Lateralis (GL) during two running strategies. This study demonstrated that both intensity and running styles impact not only muscle fatigue, but also the potential for muscle recovery during physical activity. This was observed through an increase in MDF and MNF values over time (Yousif et al., 2019b). A further example focuses on the analysis of the values of the dynamics of changes in the linear regression coefficient of the MDF parameter during walking, considering both the active phase and the rest phase. The analysis revealed a decrease in MDF values during walking, indicative of muscle fatigue. On the contrary, during the rest phase, an increase in this parameter occurred, attributed to muscle regeneration (Siecinski et al., 2022).

The current study serves as a continuation of the previous work of the authors, in which the methodology for determining the values of the MDF and MNF parameters of the linear regression coefficient was described, exemplified by a single participant in the context of muscle fatigue in selected muscles. Furthermore, a detailed review of the tools employed in spectral analysis was conducted, considering two types of movement, static and dynamic, with a focus on assessing the level of muscle fatigue. In previous work, it was emphasized that few studies describe the use of the wavelet transform, despite its acknowledged effectiveness in fatigue analysis in dynamic movement (Daniel and Malachowski, 2023) In the absence of research specifically addresses quantitative determination of fatigue during dynamic movement on a rowing ergometer, the algorithm outlined in the previous study was applied to derive fatigue parameters within a defined group. Unlike other scrutinized studies, the current investigation seeks to discern variations within the examined group of athletes. The purpose of this study is detailed as follows:

• validation of proprietary algorithm:
  - the study rigorously validated the proposed proprietary algorithm, building on the authors’ previous work. The validation process involved a practical investigation conducted on the sports section of the Academic Sports Association (AZS) at the Military University of Technology (WAT),
  - EMG signals from three major muscle groups (GAS, BF, RF) of both legs were meticulously measured during dynamic rowing ergometer exercise. Notably, athletes executed the exercise under conditions of maximum subjective fatigue,
  - consistent with the theoretical framework outlined in the previous work, the EMG signals underwent DWT analysis (Wodarski et al., 2020). Subsequently, the spectral parameters, specifically MNF and MDF, were computed based on the obtained power spectral density.
  - the linear regression equations were then derived from the MNF and MDF distributions for each muscle group. A quantitative assessment of fatigue for the selected muscle groups during the rowing ergometer exercise was performed, utilizing the linear regression coefficient,
  - based on evidence supporting the effectiveness of both MNF and MDF, and their similar behaviour in EMG signals (Phinyomark et al., 2012b), a strategic decision was made to exclude the MNF parameter from further analysis,
• statistical analysis for group homogeneity:
  - a meticulous statistical analysis was conducted to confirm the homogeneity of the studied group. This step was intended to ensure the robustness and reliability of the experimental findings,
• verification of force moments and timing on muscle fatigue index:
  - the study went beyond algorithm validation to investigate the practical implications of force moments and timing on the muscle fatigue index,
  - through systematic verification, the research explored how force moments and timing factors contribute to the overall muscle fatigue index, providing valuable information on the nuanced dynamics of fatigue during rowing ergometer exercise. The underlying hypothesis behind this analysis was to investigate whether the group remains homogeneous despite variations in strength and achievements, aiming to discern if diverse outcomes are achieved.

These purpose and contributions collectively contribute to a comprehensive understanding of the algorithm’s validity, group homogeneity, and the practical implications of force moments and timing on muscle fatigue.

## 2 Materials and methods

### 2.1 Participants

Eight healthy young volunteers (8 men) who are members of the rowing ergometer sports section (Military University of Technology Ergometer Section) were recruited for the study. The athletes were not overweight and had no history of musculoskeletal changes. The physical characteristics of the participating athletes are presented in Table 1:

**TABLE 1:** Participants characteristics.

	Mean ± SD	Minimum	Maximum
Age	21.5 ± 1	20	23
Body height (cm)	183 ± 6	175	193
Body mass (kg)	86.5 ± 8	74.8	98.2
BMI (kg/m^2^)	25.8 ± 1.89	22.75	28.82
Activity per week	4 ± 1	3	5

The median (± standard deviation) of the participants is as follows: age 21.5 ± 1 year; height 183 ± 6 cm; body mass 86.5 ± 8 kg; body mass index (BMI) 25.8 ± 1.89. Each participant is physically active and participates in physical training (gym, ergometer, running) an average of 4 ± 1 sessions per week. The participants were interviewed about their history of athletics and achievements. Of particular relevance to the study was the discernment of disparities in performance on the rowing ergometer. Consequently, the optimal times attained over a 1000 m distance are collated in Table 2. Each participant was assigned points (100 points for the fastest time, 0 points for the slowest time, with proportional values for intermediate performances) for subsequent analytical procedures and simplified data presentation.

**TABLE 2:** Comparing time results on the ergometer at a distance of 1000 m.

Participants	Times in a 1000m rowing ergometer race [s]	Scores
P1	177.9	100
P2	194.3	45
P3	194.8	43
P4	195.5	41
P5	195.9	40
P6	197.4	35
P7	205.6	7
P8	207.7	0

The frequency of consumption of potentially harmful health foods by participants is similar to the frequency of consumption of potentially beneficial health foods. Consequently, it has been determined that the diet has a neutral impact on health within the examined group. This implies that the studied cohort does not show differences in terms of overall dietary characteristics.

Participants with a history of cardiovascular disease, lower extremity pathologies, lower extremity surgeries, or neurological disorders were excluded from the study, as verified by a sports medicine physician. Participants were familiar with the measurement procedure and the possible risks of the test and confirmed their willingness to participate by giving their written consent. The study is carried out according to the decision of the Ethics Committee for Research with Human Participation of SGGW number 19/22.

### 2.2 Data collections

The entire study was conducted under standard training conditions for all participants. Participants were instructed to arrive in the laboratory in a well-hydrated and rested state, at least 3 h after eating, and were asked to avoid increased activity the day before the study (Rodrigues et al., 2022). Furthermore, following the coach’s recommendations, the meal consumed should primarily consist of carbohydrates to improve performance during exertion.

The measurement of limb torque was performed for each participant for the lower leg at the knee joint. Participants were asked to assume a specific sitting position and then secured with straps to the isokinetic dynamometer chair (Biodex Medical Systems Inc., Shirley, NY, USA) (Dejneka et al., 2022). Data on concentric flexion and extension of the knee were collected in the following ranges of motion: 30°, 60°, 90°. Participants were instructed to push the knee away as much as possible and then flex the knee toward them as strongly as possible. The purpose of this measurement was to determine the dominant leg for each participant.

The main stage of the research involved measuring the EMG activity of the muscles of the lower extremities during rowing ergometer exercise, specifically the gastrocnemius muscle (GAS), the rectus femoris muscle (RF), and the biceps femoris muscle (BF). A 32-channel Ultium EMG system (Noraxon, DTS, Desktop Direct Transmission System, Scottsdale, Arizona, USA) with a sampling frequency of 4 kHz was used for recording the EMG signal (MacEira-Elvira et al., 2019). To read the recorded data and continuously monitor the EMG signals in real time, the dedicated MyoResearch XP Master Edition system was applied. According to SENIAM standards, surface electrodes made of Ag/AgCl material were placed on the skin with a 2 cm spacing between them. Before electrode placement, the skin was shaved and thorough cleaning with a mixture of alcohol and ether was performed to minimize impedance. An important aspect of signal registration was the positioning of the participant’s foot, distinguishing the work of individual muscle groups based on the pressure on the ergometer base.

**FIGURE 1:**
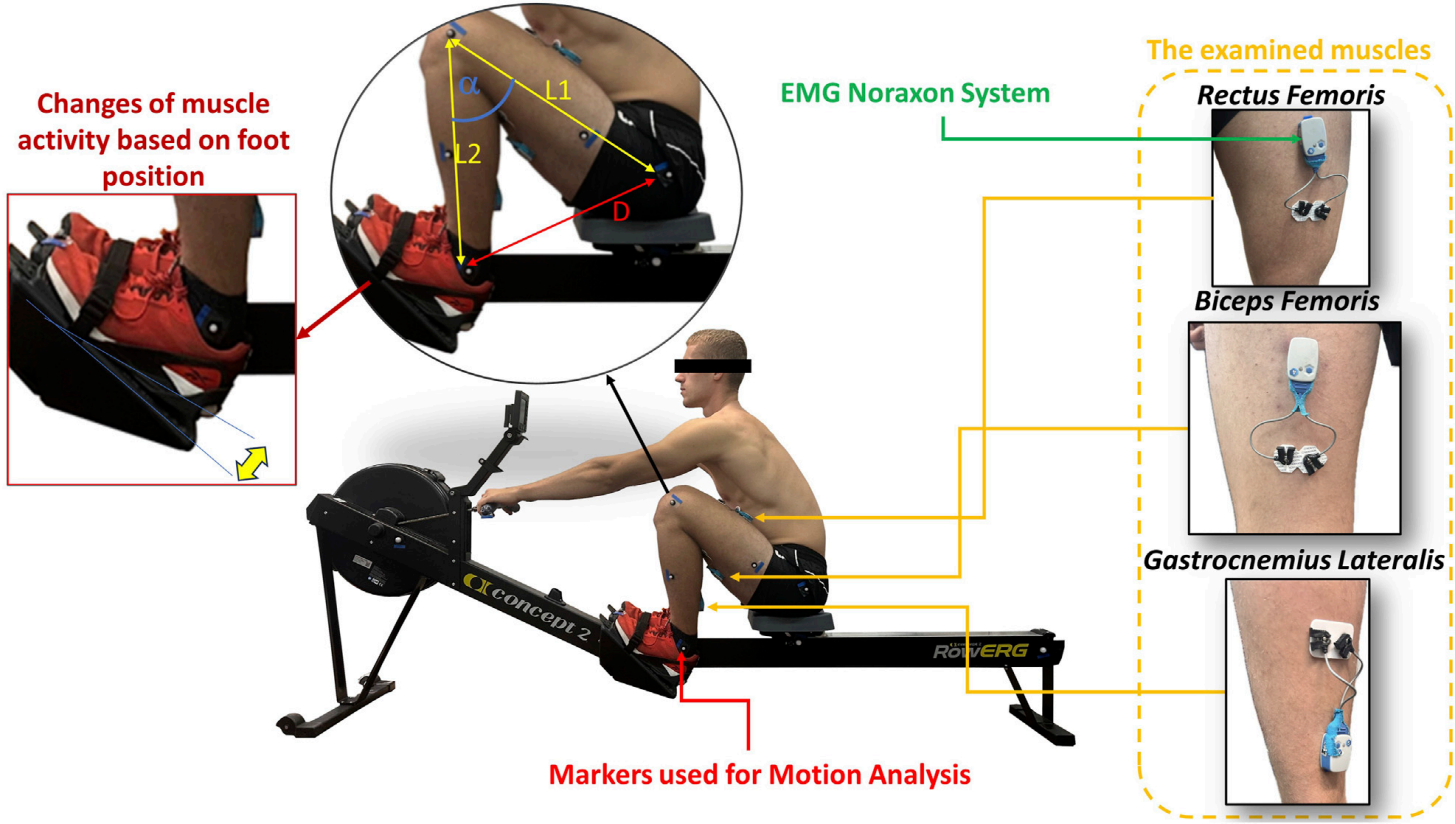
The object of study along with the specified equipment, the muscles examined, and the measurements details.

During the EMG measurements, changes in the position of the lower extremity were recorded using the Myo Video software, which is part of the Noraxon system. The task of the software was to register the location of the markers in individual video frames, and with the use of calibration data, measurements were obtained, including the distance between them (Joel Martin and St Andrews Crossfit Nittany, 2012; Daniel and Malachowski, 2023). Record changes in the position of the lower extremity to determine the knee flexion of each participant. Therefore, markers were placed, among other areas, at the ankle, knee, and hip. The setup of the measurement apparatus in the starting position is illustrated in Figure 1.

The initial scenario involved the angle of flexion of the direct measurement of the knee as the angle between segments L1 and L2, facilitated by the MyoVideo software. Due to the movement characteristics of the rowing ergometer for some participants, leading to the obstruction of the knee marker by their hand, a direct measurement of the angle was not feasible. Instead, an indirect measurement of the angle was conducted by monitoring the distance between markers placed on the participant’s ankle and hip as a function of time. Additionally, before the test, the segmental lengths of the lower extremities of all participants were measured. Table 3 presents the results of the anatomical measurements, which are absolute values measured from the hip to the knee (L1) and from the knee to the ankle (L2).

**TABLE 3:** Comparison of distances between significant markers.

Participant	Distance between hip and knee - L1 [mm]	Distance between knee and ankle – L2 [mm]
P1	475	445
P2	485	490
P3	520	485
P4	490	465
P5	465	460
P6	455	485
P7	440	435
P8	445	440

The collected data allow the calculation of the knee flexion angle based on the formula derived from the law of cosines (graphic visualization of this case in Figure 1.):

$$\alpha =\arccos\;\left(\frac{L_{1}^{2}+L_{2}^{2}-D^{2}}{2L_{1}L_{2}}\right)$$

where:

- $L_{1}$ – distance between hip and knee,
- $L_{2}$ – distance between knee and ankle,
- D – distance between hip and ankle.

Before starting the experiments, subjects were asked to warm up on a 500 m indoor rowing machine (Model E, Concept II, Morrisville, NC, USA) (Joel Martin and St Andrews Crossfit Nittany, 2012) to get used to the rowing machine. Subsequently, they rested for 10 min, during which the measurement devices were verified. After confirming the functionality of the measurement equipment, participants were instructed to assume the starting position and initiate movement upon the first auditory signal. They then proceeded to perform the test, which involved rowing on the rowing ergometer to the rhythm of the sound signal (30bpm). In the first minute of the exercise, participants set the resistance on the rowing ergometer at level 4, in the subsequent minute to level 3, then 2, and in the fourth minute to level 1. At level 1 resistance, participants continued rowing until reaching a state of maximal fatigue.

During movement, participants were corrected based on the previous analysis of their rowing technique in terms of common errors such as leg straightening or pulling hands towards the rowing machine. During the exercise, participants were asked about their level of fatigue each minute using the Borg Rating of Perceived Exertion (RPE) scale (Daniel et al., 2023). RPE is based on the physical sensations experienced by an individual during physical activity, including increased heart rate, increased breathing or breath frequency, increased perspiration, and muscle fatigue. Although it is a subjective measure, the evaluation of exertion, based on a scale ranging from 6 to 20, can provide an estimate of muscle fatigue during physical activity (Williams, 2017). In this study, the scale was used to determine the end of the exertion, with the participants reporting a maximum level of 20.

### 2.3 Data processing

**FIGURE 2:**
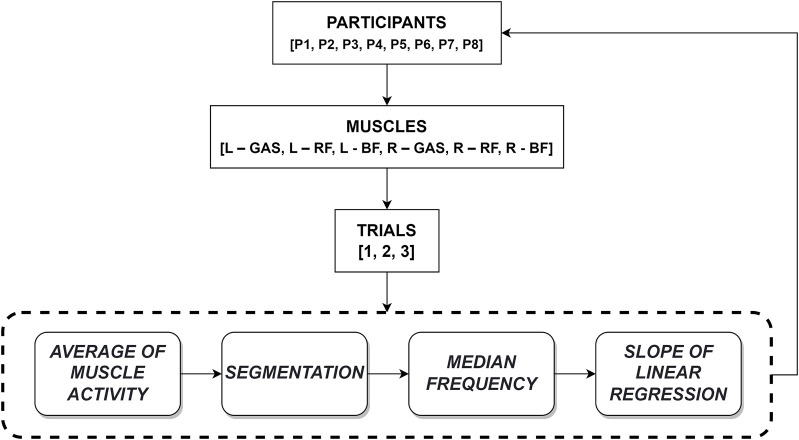
Data processing algorithm.

To perform the EMG signal analysis, data was collected during tests involving eight participants. Each participant performed three tests, and in each trial, EMG signals from six muscles were recorded: L–GAS, R–GAS, L - RF, R–RF, L–BF and R–BF. Each EMG signal was pre-processed by applying a Butterworth filter with cutoff frequencies of 20 Hz and 500 Hz to delimit the physiological frequency band of the EMG signal and to remove high- and low-frequency interference (Daniel et al., 2023). Data processing was carried out based on the diagram presented in Figure 2.

**FIGURE 3:**
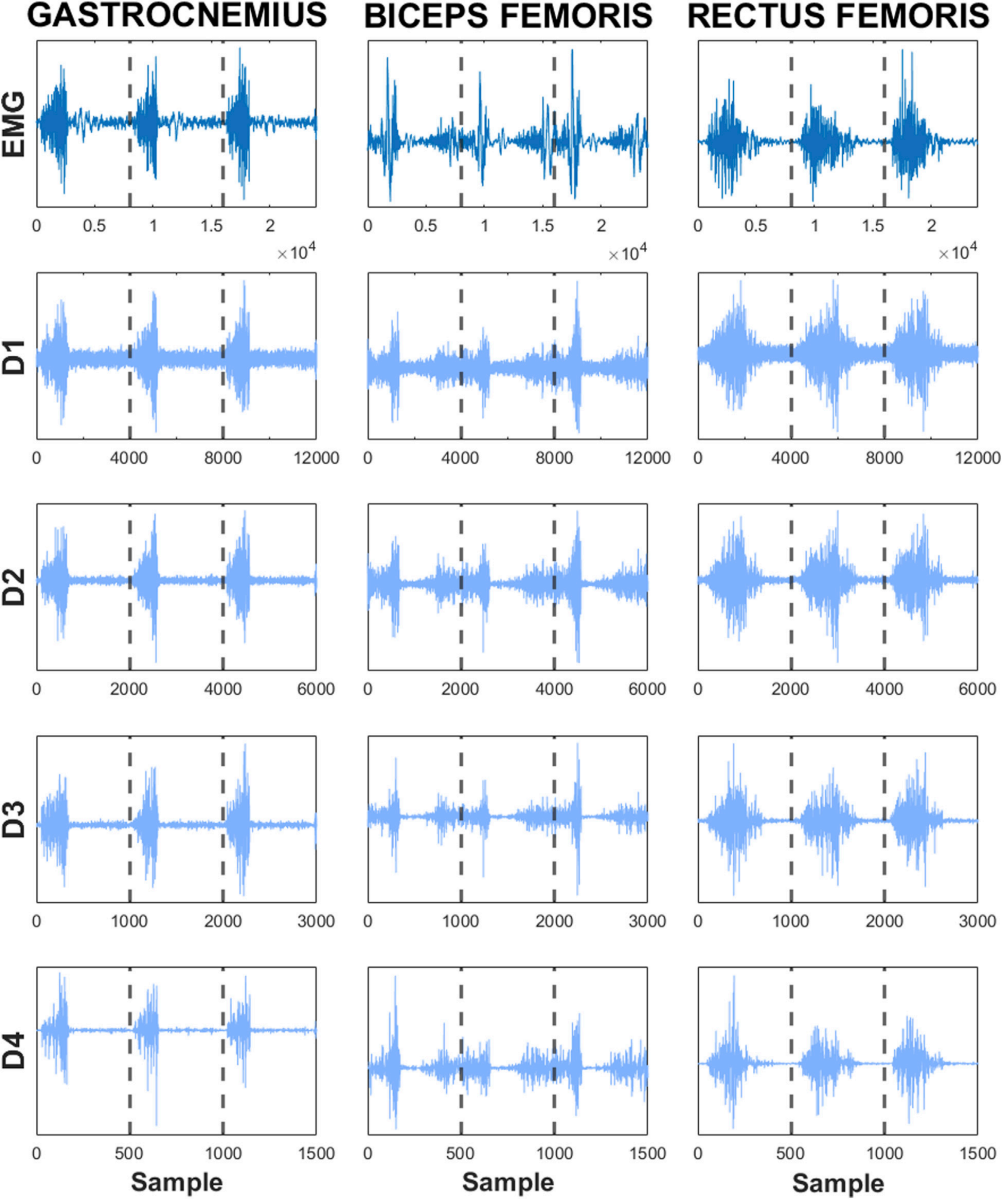
Analysis of DWT for segments of EMG signals from selected muscles (GAS, BF, RF). The details: D1, D2, D3, D4, Original EMG signal: EMG.

Based on the determined function of the changes in the knee flexion angle and the EMG signal, the average muscle activity was calculated. At the moments when the knee flexion angle function reached a minimum value, the boundary between adjacent segments was identified. Based on this, the EMG signal was divided into segments lasting approximately 2 s (2 ± 0.082 s), covering a complete, single cycle performed by the participant. The segmentation of the EMG signal allowed for DWT analysis for each cycle of activity, enabling the determination of MDF as a frequency parameter. Wavelet transformation is a spectral estimation technique that represents the signal as a linear combination of functions obtained through transformations, such as scaling and translation, based on mother wavelets. In this work, the Daubechies4 mother wavelet was used due to its effectiveness in analyzing the power spectrum of the EMG. To determine the mentioned frequency parameter, the signal was decomposed, resulting in detailed and approximation coefficients. The decomposition process involves a series of operations on low-pass and high-pass filters, as well as down-sampling. In Figure 3, an example DWT analysis of EMG signal segments from three different muscles of a selected participant is illustrated.

Subsequently, for signal reconstruction, the reverse decomposition process was applied using a synthesis filter. On the basis of the obtained wavelet coefficients, MDF values for a single cycle of activity were determined. For each EMG segment, one MDF value was obtained. Using the entire range of the EMG signal, it was possible to calculate the slope coefficient of the linear regression of the MDF variable. Wavelet analysis, determination of MDF values, and calculation of the slope coefficient of the linear regression of the MDF variable were performed using Matlab software.

**FIGURE 4:**
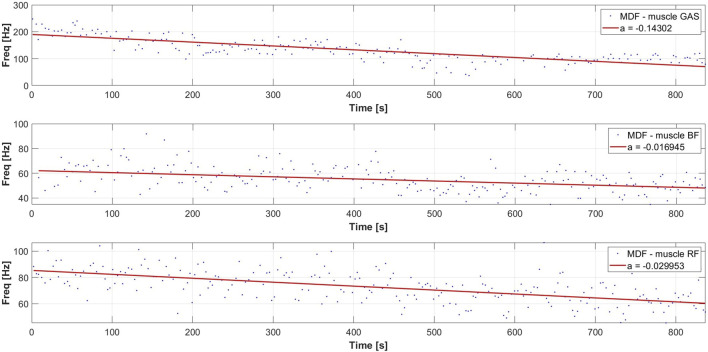
Typical distribution of the MDF parameter and linear regression of MDF for a selected participant during a rowing ergometer test for the dominant leg.

**FIGURE 5:**
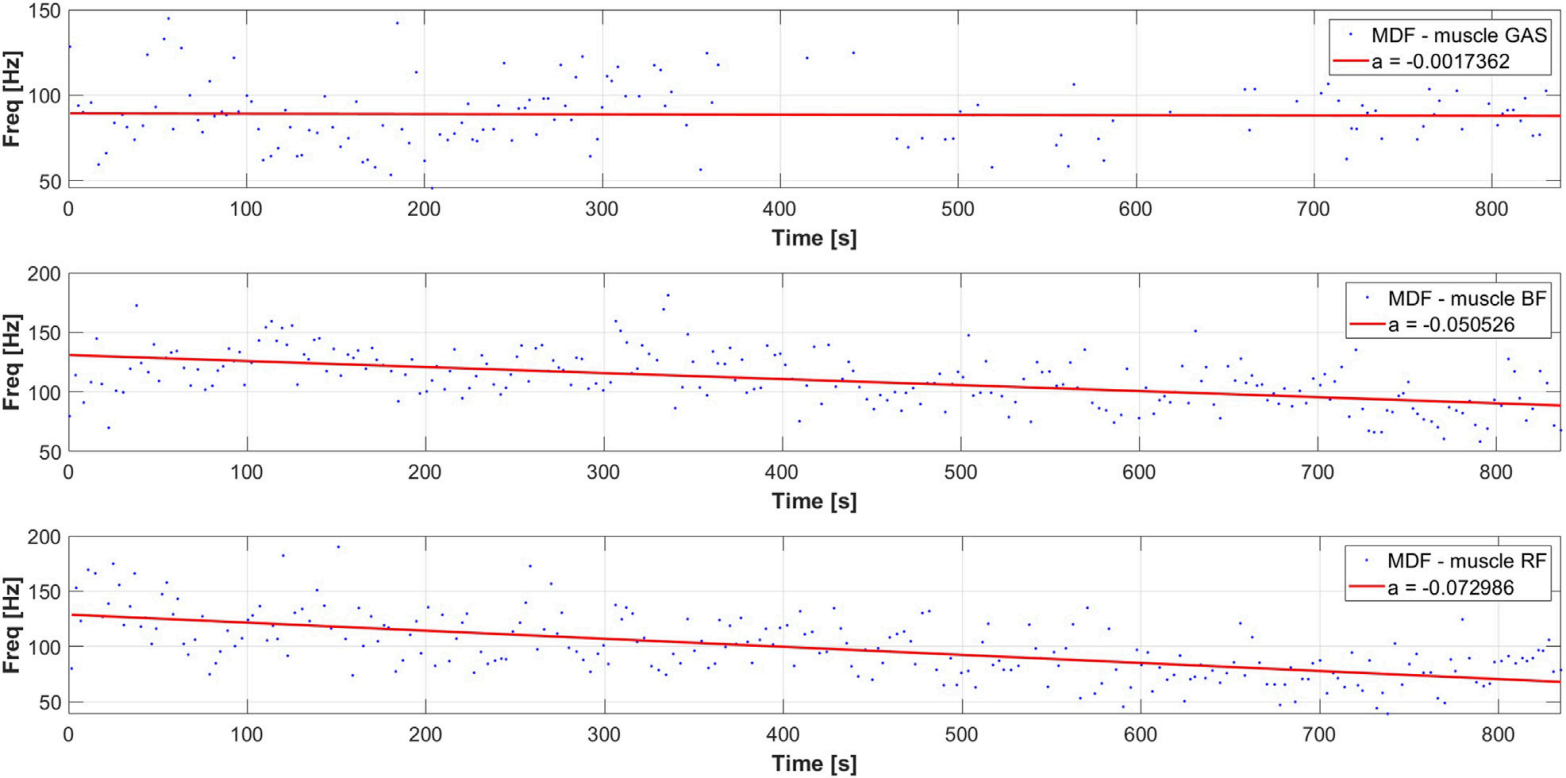
Typical distribution of the MDF parameter and linear regression of MDF for a selected participant during a rowing ergometer test for the nondominant leg.

The analysis of the EMG signal conducted resulted in obtaining a set of linear regression coefficients for the MDF variable for each participant, allowing for a series of statistical analyses. The distribution of MDF for each of the dominant leg muscles of one of the participants is presented accordingly in Figures 4, 5, along with the calculated slope coefficient of the linear regression of these variables. The decrease in MDF over the duration of the exercise indicates an increase in the degree of muscle fatigue.

### 2.4 Statistical analysis

The characteristics of the participants and the measured measurements analyzed were used for the descriptive statistics performed using OriginPro 2023 software (OriginLab Corporation). To assess the normality of the distribution, the Shapiro-Wilk test was used with a significance level set at *p* < 0.05. Data were presented as median ± interquartile range. Furthermore, a statistical test was performed that compared the mean muscle fatigue parameters between the dominant and non-dominant legs using the Mann-Whitney test, with a significance level set at 0.05.

## 3 Results

The regression results for the muscle group, including GAS RF and BF were calculated during the rowing ergometer test under the same conditions described in the Materials & Methods section. The values obtained from the wavelet analysis for each muscle during each trial were analyzed in two analyses. The first analysis focused on the times’ influence achieved at 1000 m distance on the fatigue index, while the second analysis aimed to examine the impact of the force moment of each participant on the level of muscle fatigue.

### 3.1 Analysis of fatigue data for rowing ergometer exercise and relationships with previous rowing performance results

As muscle fatigue allows for the assessment of overall body fatigue, it was decided to average the fatigue results for all muscles in all trials (Chang et al., 2012). The muscle fatigue data tested in each trial for each person did not exhibit a normal distribution in all tests. Table 4 presents the results of the normality test.

**TABLE 4:** Result of the normality test.

Participant	Statistic	*p*-value	Decision at level (5%)
P1	0.95194	0.66885	Can’t reject normality
P2	0.83504	0.0064	Reject normality
P3	0.87431	0.02574	Reject normality
P4	0.81389	0.00317	Reject normality
P5	0.94205	0.52505	Can’t reject normality
P6	0.8175	0.01492	Reject normality
P7	0.95206	0.45825	Can’t reject normality
P8	0.93899	0.27846	Can’t reject normality

**FIGURE 6:**
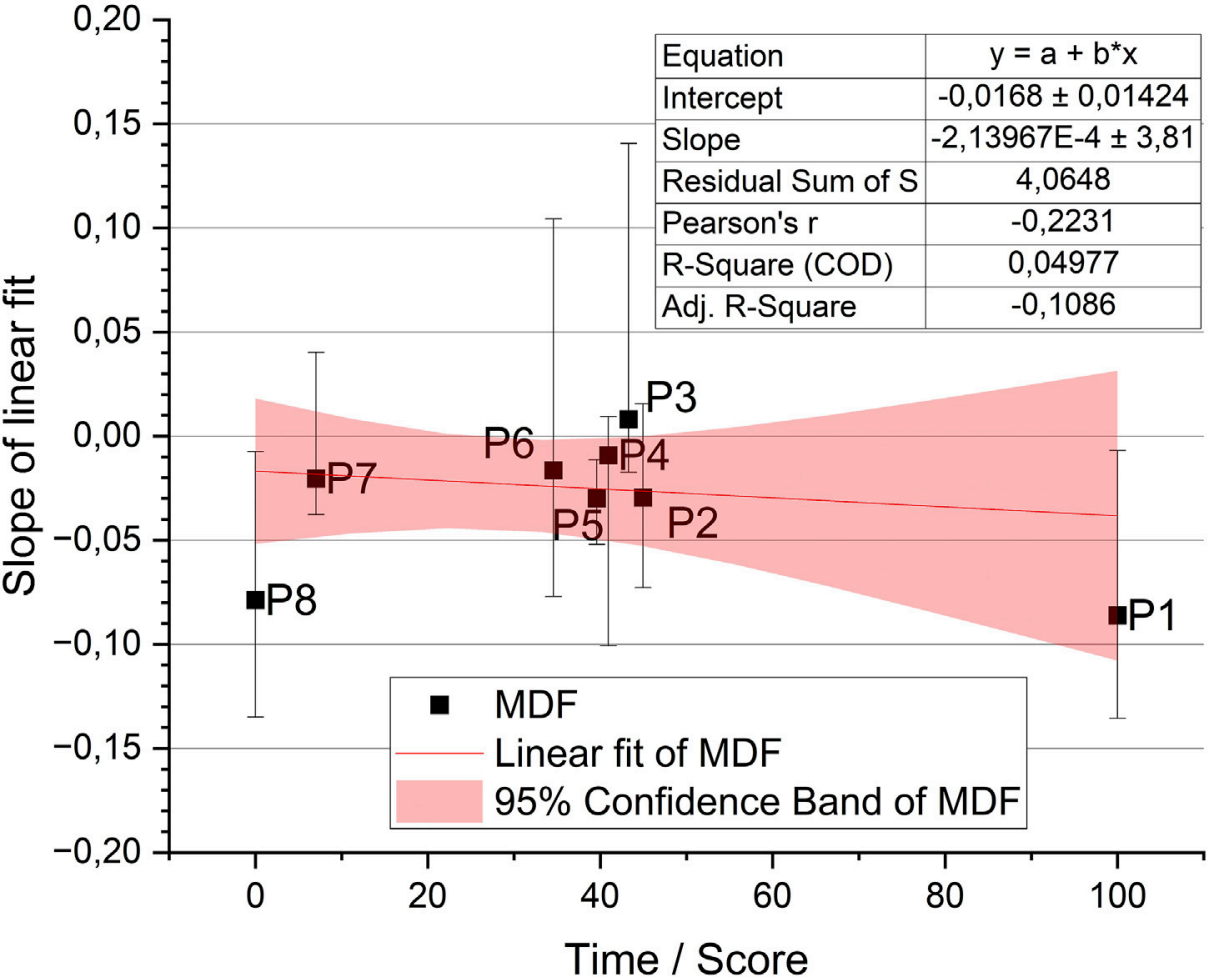
Distribution of the MDF parameter for all participants according to their achieved scores (Table 2) on the rowing ergometer.

The results of the normality test indicate that tests based on normal distributions cannot be applied to compare the data. Therefore, the data are presented in the form of median and interquartile range in Figure 6.

As part of the conducted study, participants (P1-P8) were classified according to the results achieved, according to the data presented in Table 2. The median values obtained for all participants (except P3) are characterized by negative values, consistent with the findings of the scientific literature, suggesting the appearance of muscle fatigue due to the physical effort taken. Furthermore, a similarity can be observed in the median fatigue values between the participant with the best time (P1 = −0.08615) and the participant with the worst time (P8 = −0.07872). The median distribution of the remaining participants shows no correlation between the achieved times and the level of muscle fatigue (P2 = −0.02946, P3 = 0.00804, P4 = −0.00927, P5 = −0.02979, P6 = −0.01644, P7 = −0.02043). To analyse the differences between groups of participants, the interquartile range was applied. On this basis, no differences were observed with respect to the test conducted for this specific group of participants.

Based on the analysis conducted, an ANOVA test was performed to determine the relationship between the previous ergometer performance of the participants (Table 2) and muscle fatigue. One-way analysis of variance (ANOVA) was used to compare the means of muscle fatigue levels in different time achievement groups. Specifically, the participants were categorized based on the results presented in Table 2. The test results are presented in Table 5.

**TABLE 5:** The result of the ANOVA test in the case of studying the relationship between time achievement and muscle fatigue.

		Sum of Squares	Mean Square	F Value	Prob>F
Median	Model	0.21291	0.21291	0.31427	0.59537
	Error	4.0648	0.67747	-	-
	Total	4.27771	-	-	-

The results of the ANOVA include information on the F-statistic, degrees of freedom, and *p*-value. It is important to note that the ANOVA test was chosen as it allows for the comparison of means among multiple groups simultaneously. The significance level was set at 0.05 to determine whether there were statistically significant differences in muscle fatigue levels between participants with different time achievements.

### 3.2 Analysis of data on the dominant and non-dominant leg. The relationship between the strength level of the dominant leg and muscle fatigue

From the measurements on the isokinetic dynamometer, the force moment values were obtained at positions of 30°, 60°, and 90° during knee extension for both limbs. Subsequently, the average values within each limb were calculated, and the higher of these values was used to determine the dominant leg. This value was also considered in the further analysis. Table 6 presents the complete results of the force moment measurements, and the points were assigned in a manner analogous to the participants’ achieved times.

**TABLE 6:** Comparison of force results.

Participants	The force moment for the dominant leg [Nm]	Scores
P1	268.2	69
P2	303.7	100
P3	296.3	94
P4	290.9	89
P5	291.6	89
P6	242.3	46
P7	262.2	63
P8	190.3	0

Despite the results of the dynamometer indicating which leg is dominant, the decision was made to analyse the fatigue results for both legs, taking into account the established dominant and non-dominant character for each limb. For this purpose, it was checked, using the normality test, that the data did not exhibit such a character. Subsequently, the Mann-Whitney test was conducted, which did not confirm significant differences between dominant and non-dominant legs in terms of their impact on muscle fatigue.

**FIGURE 7:**
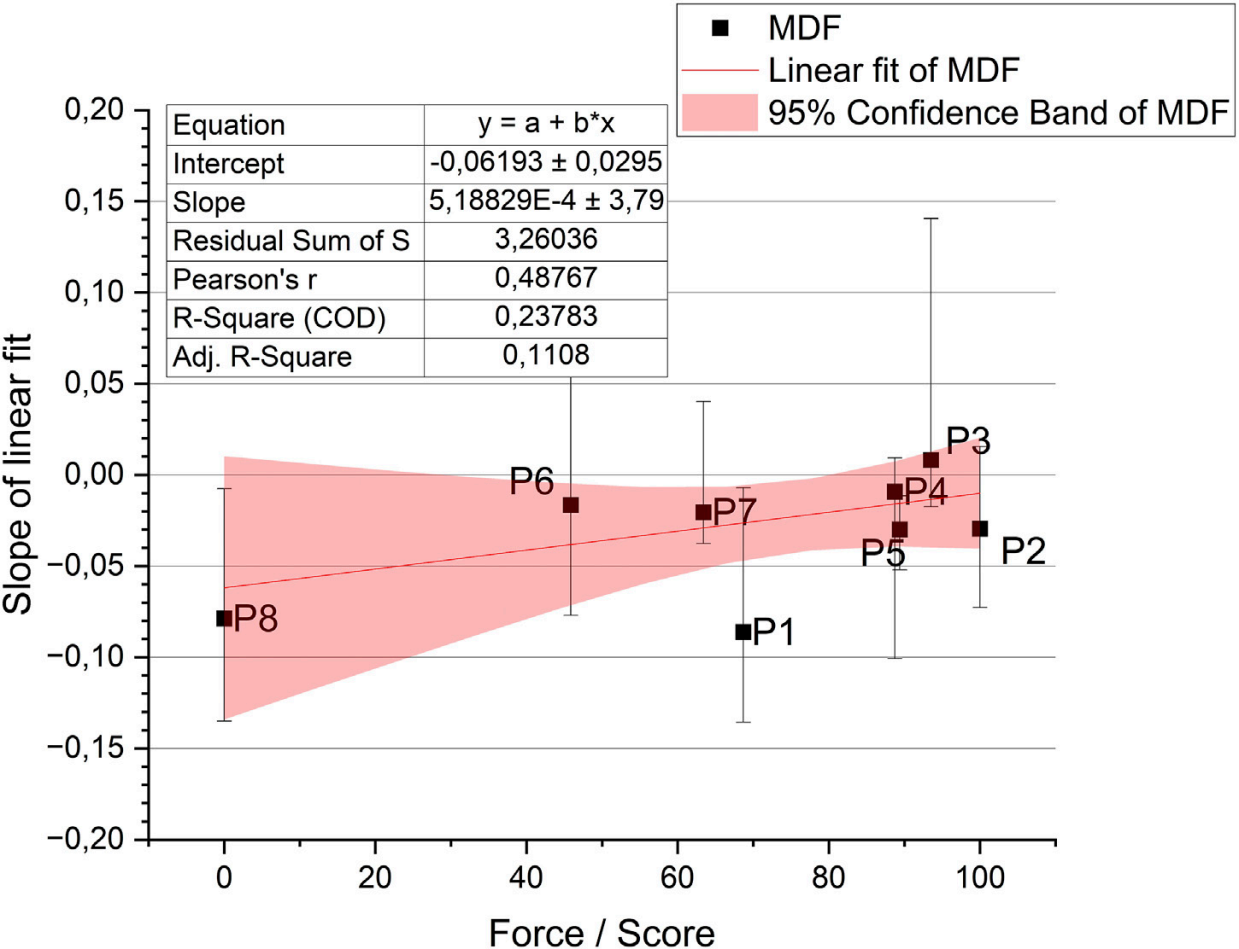
Distribution of the MDF parameter for all participants according to the force score (Table 6).

Despite the lack of identified differences between the legs, the relationship between fatigue for all muscles (linear fit slope) of the dominant leg and the moment of force was analyzed (Table 6). Data depicting the relationship between force and fatigue are presented in Figure 7.

Similarly to the previous subsection, an ANOVA test was performed to determine the relationship between fatigue and the moment of force. One-way analysis of variance allowed for the comparison of means across different force levels. The results of the ANOVA test are presented in Table 7, which provides information on the F-statistic, degrees of freedom, and the *p*-value. The chosen significance level was 0.05. This analysis aimed to determine whether there were statistically significant differences in muscle fatigue levels related to different force levels.

**TABLE 7:** The result of the ANOVA test in the case of studying the relationship between forces and muscle fatigue.

		Sum of Squares	Mean Square	F Value	Prob>F
Median	Model	1.01735	1.01735	1.87221	0.22025
	Error	3.26036	0.54339	-	-
	Total	4.27771	-	-	-

The test conducted indicates that the slope of the line in both cases is not different from zero, meaning that there is no statistical evidence of the impact of time or force on fatigue. Based on this, one can conclude that the level of fatigue does not depend on the results achieved by the participants on the rowing ergometer.

## 4 Discussion

Muscle fatigue is considered in both static and dynamic movements, employing various advanced signal processing techniques. Furthermore, research on the quantitative determination of fatigue is conducted in comparative group settings, such as between genders (female-male) and different levels of fitness (trained-untrained individuals). In addition, these studies often focus on a single muscle (or muscle group) and typically involve a single measurement repetition. They also include various sports disciplines. Consequently, the decision was made to propose an analysis and confirm homogeneity as a basis for subsequent comparative research and communication. For the current study, the authors selected participants from the student sports section of AZS WAT, who, as military students, share a similar lifestyle. Each participant had participated in sports competitions and their results were consistently ranked, allowing their arrangement from the least to the most trained athletes. The authors conducted a series of tests to assess their homogeneity, exploring the impact of diet on health, general characteristics, history of injuries, and history of sports activity. The participants underwent a test involving maximum subjective effort at a rhythm of 30 bpm. This form allowed determination of angle changes, muscle activation and deactivation, and subsequently, the segmentation of the signal. In addition, the measurements were performed under controlled conditions, including participant preparation, test procedure, and real-time signal monitoring, all contributing to the quality of the results obtained.

The authors of the study operated under the assumption that muscle fatigue translates into overall bodily fatigue (Chang et al., 2012). Consequently, they decided to perform calculations for all tests and muscles simultaneously. Regression coefficients were determined for each muscle, each trial, and each participant. One of the main objectives of the study was to verify whether the results obtained using the algorithm developed in previous work (Daniel and Małachowski, 2023) align with the actual occurrence of muscle fatigue, resulting in a decline in MDF values over time. In some cases, positive regression coefficients for MDF were observed, which may be due to various limitations. One such limitation is the individual characteristics of the participants, which inherently vary among them (for example: displacement and modification of the volume of the muscle being analysed influence on EMG measure (Romero and Gual, 2010) or sweating during the long duration of exercise (Bala and Joshi, 2022)). Furthermore, distortions and interference of the sEMG signal, which can result from, e.g., crosstalk of EMG signals of adjacent muscles, cannot be eliminated during the data analysis phase, which can also affect these results (Zhang et al., 2022). In dynamic movement, changes in sensor positioning may occur and cause sEMG signal artifacts (Perpetuini et al., 2023), which adds an additional layer of limitation to this type of measurement. Moreover, investigations into the prediction of fatigue-induced electromyographic signals are also of interest (Bala and Joshi, 2022). One study explored this by analyzing initial sEMG recorded over a shorter time period during bicep flexion with a load. Another important area of focus is the investigation of stimulation patterns based on muscle synergy, which holds the potential to decrease muscle fatigue (Bala et al., 2023). However, when applied to dynamic movements, this approach may require the consideration of additional factors. Nevertheless, this aspect presents an intriguing point for future research in the field.

Subsequently, data analysis was performed to assess individual muscle fatigue or specific trials; however, no significant differences were found between the fatigue parameters analyzed. This lack of significance may be due to various factors. One limitation in this case could be the sample size of the studied data or incomplete input data (lack of data from the third measurement for three participants).

To assess whether other factors, such as torque or times achieved over a 1000 m distance, influence muscle fatigue, a data distribution analysis was conducted. The analysis did not indicate that such dependencies exist. However, a limitation in this case may be that torque measurement was performed in a static context, while the activity on the ergometer is dynamic. Monitoring torque values during movement would be beneficial for future research.

The practical application of the conducted research could be implemented in the future by modifying the measurement conditions to more accurately assess fatigue. Additionally, the utilization of such data could serve as a reference point not only for achieving fatigue levels on a rowing ergometer, but also for conducting and comparing results in other types of dynamic movements.

The present study is innovative in testing both the algorithm developed and analyzing the studied group regarding the distribution of data related to the MDF linear regression coefficients. Additional tests were used to determine whether other factors might influence the trend in the subjects’ data (e.g., whether a person with the lowest MDF regression coefficient is associated with their ranking or whether someone with the highest torque value exhibits the lowest quantitative fatigue value). As these analyses did not reveal such dependencies, the current group is considered homogeneous. The authors found this result satisfactory, as the presented findings will be utilized in a future study involving virtual reality (VR).

## 5 Conclusion

The method utilized to calculate fatigue using wavelet analysis in the study group of rowers from AZS WAT appears to be effective in assessing muscle fatigue during dynamic rowing exercises. Taking into account the proposed methodology, conducted tests, and results obtained, the following conclusions have been formulated:

• Wavelet analysis of sEMG presents a promising approach to quantitative assessment of muscle fatigue during rowing ergometer exercises, as evidenced by the achievement of negative regression values in nearly all cases examined.
• The obtained results suggest diversity in the assessment of muscle fatigue, encompassing the muscles studied, trial variations, and individual participants.
• The influence of diet, injury history, and individual characteristics of participants can impact the assessment of muscle fatigue, which is a significant limitation.
• Despite attempts to analyse the relationship between force moments and muscle fatigue, the current study did not reveal significant correlations in the examined group.
• The results presented lay the foundations for future research, particularly in the context of using virtual reality to analyse muscle fatigue during rowing ergometer exercises. The hypothesis for future research involves determining the impact of using virtual scenery on fatigue. Therefore, the present study examined other potential factors that could influence the homogeneity of the group.

## Data Availability

The raw data supporting the conclusion of this article will be made available by the authors, without undue reservation.
